# 

**Published:** 1881-07

**Authors:** 


### CONTENTS.

| | Page. | | Page. |
|---|---|---|---|
| Signs of Disease | 241 | Mental Epidemics | 257 |
| Abolish the Use of Liquors | 243 | Dangerous Newspaper Receipts | 260 |
| Spanish Murders and Brigandage | 244 | Poisons | 264 |
| Taking Comfort in Life | 245 | Debt and Death | 265 |
| The People Angry | 246 | Characteristics of Doctors and Others | 267 |
| How to Get Along | 247 | Yankees in Russia | 269 |
| Truffles | 248 | Youth and Age (poetry) | 270 |
| The Dwarf Diplomat | 249 | Origin of Diphtheria | 271 |
| The Winning of Wealth | 250 | How to Hang Pictures | 272 |
| Treatment of Women | 250 | Power of the Plug Hat | 272 |
| Our Friends Beyond | 252 | A New Fashion of Sleeping | 273 |
| The Nursery Elf (poetry) | 252 | The Saragossa Sea | 274 |
| Natural Death | 253 | Major Andre's Remains | 275 |
| Our Changing Climate | 253 | Women with Moustaches | 275 |

